# Effect of methionine and trace minerals (zinc, copper and manganese) supplementation on growth performance of broilers subjected to *Eimeria* challenge

**DOI:** 10.3389/fphys.2022.991320

**Published:** 2022-11-21

**Authors:** J. Chen, F. Yan, V.A. Kuttappan, K. Cook, B. Buresh, M. Roux, D. Hancock, Mercedes Vázquez-Añón

**Affiliations:** Novus International Inc., St. Charles, MO, United States

**Keywords:** chelated trace minerals, total sulfur amino acids, coccidiosis, broiler, ITM, TBCC, methionine, vaccination

## Abstract

Coccidiosis is a major intestinal challenge that causes economic loss to the broiler industry. Two battery cage studies were conducted to evaluate the effect of trace minerals, source and dose of methionine on growth performance and gut health of broilers subjected to *Eimeria* challenge. Experiment #1 consisted of 9 treatments of 2 × 2 × 2 factorial design + 1 arrangement with main factors of methionine (Met) sources (DL-Met vs*.* 2-hydroxy-4-(methylthio)-butanoic acid (HMTBa)), total sulfur amino acid (TSAA) levels (high vs*.* low; ±5% of recommended level), and sources of trace minerals (TM) Zn:Cu:Mn in the form Inorganic trace minerals (ITM) in sulfates (80:20:100ppm) vs*.* mineral methionine hydroxy-analogue bis-chelate (MMHAC, 40:10:50ppm), each with 8 pens of 10 birds. Experiment #2 consisted of 2 treatments--ITM [ZnSO_4_:tribasic copper chloride (TBCC):MnSO_4_ 110:125:120ppm] and MMHAC (Zn:Cu:Mn, 40:30:40ppm), each with 36 pens of 10 birds. All birds except for treatment 9 in experiment #1 were orally gavaged with 1x, 4x and 16x recommended dose of coccidiosis vaccine on d0, d7 and d14, respectively. Data were subjected to one-way and/or three-way ANOVA, and means were separated by Fisher’s protected LSD test with significance at *p* ≤ 0.05. In experiment #1, factorial analysis revealed the main effects of TSAA level and TM, but not Met source. High TSAA level improved body weight and cumulative feed intake at 14, 20, and 27d. MMHAC improved body weight at 14, and 27d; feed intake at 14, 20, and 27d; and cumulative FCR at 27d vs*.* sulfates. One-way ANOVA analysis showed that birds fed MMHAC and high levels of TSAA regardless of Met source had similar body weight as unchallenged birds on d27. In experiment #2, MMHAC improved body weight and cumulative FCR, and reduced jejunal IL-17A gene expression on d28. In summary, in broilers subjected to *Eimeria* challenge, supplementation of the reduced levels of bis-chelated trace minerals MMHAC improved growth performance compared to high levels of ITM (sulfates or TBCC), which might partially result from better immune response, high levels of TSAA improved growth performance, Met source had no effect. Supplementation of both bis-chelated trace minerals MMHAC and high levels of TSAA could overcome the growth performance challenge issue due to coccidiosis.

## Introduction

Coccidiosis is one of the most prevalent enteric diseases caused by protozoan parasite of the *Eimeria* (*E.*) genus in global modern poultry production. The global cost of coccidiosis including losses during production and costs for prophylaxis and treatment in chickens is estimated to have been ∼ £10.36 billion at 2016 prices, which is equivalent to £0.16/chicken produced (Blake et al., 2020). The use of antibiotics as growth promoters in animal feed has been recognized to contribute to the emergence of antibiotic-resistant bacteria, which could be transferred to humans reducing the sensitivity of human disease treatment with certain drugs (Lekshmi et al., 2017; Tang et al., 2017). Consumer concerns have resulted in the restricted use as antibiotic growth promoters (AGP) in animal feed in certain countries to prevent the spread of antibiotic resistance. In the US, all medically important antibiotics have been banned for growth promotion since 2017, and they can only be used in animal feed or water for the therapeutic purposes to treat, control or prevent diseases under the supervision of a licensed veterinarian (FDA, 2012; FDA, 2013; FDA, 2015). Although non-medically important antibiotics are still allowed to use in animal feed, some restaurant chains and retailers chose to only use antibiotic-free poultry products, which drives poultry producers toward AGP free production in the US. The common challenges of AGP free production include the increased susceptibilities to enteric diseases such as coccidiosis and necrotic enteritis, and reduced overall poultry health, growth and immune function (Bean-Hodgins and Kiarie, 2021). Nutritional prevention or intervention is an integral part of a holistic approach to maintain a healthy gut and prevent enteric diseases to help birds reach their full genetic potential in AGP free poultry production. For coccidiosis, the poultry industry relies heavily on prophylactic in-feed anticoccidial drugs to suppress the infectious cycle and prevent coccidiosis outbreaks (Chapman, 2009), vaccination programs, or a bio-shuttle program that involves a combination of vaccination and anticoccidials (Peek and Landman, 2011; Chapman and Jeffers, 2014), however, vaccination could lead to reduced growth performance (Li et al., 2005). In recent years, more and more poultry producers are looking into natural approaches to control coccidiosis, such as essential oils, probiotics, prebiotics, trace minerals, and other gut health enhancing products, which could be complimentary to current coccidiosis control programs.

Zinc (Zn), copper (Cu) and manganese (Mn) are important trace minerals involved in many physiological functions of the body and play important roles in animal growth, development, and health. Zn is critical in a wide variety of process such as cell proliferation and animal growth, immune development, reproduction, gene regulation and defense against oxidative stress (Richards et al., 2010). Cu is widely used at high levels as growth promoter in poultry due to its well-recognized anti-microbial effect (El Sabry et al., 2021). Mn is essential for animal growth and reproduction (Underwood and Suttle, 1999), and bone development (Fawcett, 1994). Dietary Mn deficiency increased intestinal permeability by impairing intestinal tight junctions and Mn supplementation enhanced intestinal barrier and splenic inflammatory response to fight against *Salmonella* infection in broilers (Zhang et al., 2020). Cu requirements were reported to be higher for chickens experiencing an acute phase response or pathological challenge than healthy chickens (Koh et al., 1996). The chicks infected with *E. tenella* had significantly elevated levels of serum copper and ceruloplasmin and liver copper than their pair-fed counterparts (Richards and Augustine., 1988). In previous studies, mineral methionine hydroxy-analogue bis-chelate (MMHAC) was reported to improve adaptive and innate immune response in broilers and gilts (Richards et.al., 2010; Bun et al., 2011), and maternal supplementation of Zn methionine hydroxy-analogue chelate (Zn-MHAC) in breeders reduced intestinal inflammation in progeny chicks (Li et al., 2015). These findings highlight the possibility of trace minerals (Zn, Cu, and Mn) in helping manage negative impact of coccidia in the gut.

Methionine (Met) is the first limiting amino acid in all poultry corn-soybean based diets. Metabolism of sulphur amino acids generate taurine, which is known as a strong antioxidant and the most abundant free amino acid in lymphocytes, reducing the production of proinflammatory cytokines and prostaglandin E2 (Surai et al., 2021). Taurine protects gut function by regulating mucosal barrier function and ameliorating LPS-induced duodenal inflammation in chickens (Xiao et al., 2018). 2-hydroxy-4-(methylthio)-butanoic acid (HMTBa) was reported to be more effective than DL-Met in protecting gut barrier function of Caco-2 cells from exposure to oxidative stress or inflammation challenges through the metabolites produced in the trans-sulfuration pathway (Martín-Venegas et al., 2013). Both HMTBa and DL-Met could exhibit antioxidant capacity in broilers (Wang et al., 2019) and HMTBa was reported to be more efficient than DL-Met in alleviating oxidative stress in broilers under heat stress (Willemsen et al., 2011). Adequate or marginal excess of dietary HMTBa reduced inflammation in female broiler chickens subjected to LPS challenge (Matsushita et al., 2007). Supplementation of 0.75% Met in broilers fed low crude protein diet and challenged with a mild coccidia infection increased the severity of coccidiosis, indicating the negative impact of Met supplementation on coccidiosis when birds were fed low crude protein diet (Teng et al., 2021). However, there is also some research showing that the requirements of total sulfur amino acids (TSAA), Met + cysteine (Met + Cys), may be higher when chickens are infected with *Eimeria* species (Southern and Baker, 1982; Ren et al., 2020).

The digestible TSAA for starter and grower diet in broilers are 0.95% and 0.87% according to breeder recommendation and 0.94% and 0.87% according to Agri Stats. To determine whether adding higher levels of TSAA will help broilers overcome cocci challenge, 5% higher (105% of recommendation) and 5% lower (95% of recommendation) than recommended levels (100% of recommendation, 0.94% TSAA in starter diet and 0.86% TSAA in grower diet) were tested in broilers subjected to *Eimeria* challenge in one of the experiments. In addition, the effect of trace minerals and source of methionine on growth performance and gut health of broilers under coccidia challenge were also determined in the experiments.

## Materials and methods

### Birds and housing

Guide for the Care and Use of Agricultural Animals in Research and Teaching (FASS, 2010) was followed for housing and care of the animals throughout the experiment. All research procedures were reviewed and approved by the animal ethics committee composed of members from Novus International Inc (20 Research Park Drive, St. Charles, MO 63304) and a licensed veterinarian from Bridgeton Animal hospital (3148 McKelvey Road, Bridgeton, MO 63044). Ross 308 male broilers were purchased from a local hatchery (Stover Hatchery, Stover, MO). Upon arrival, birds were placed immediately in 72 pens of 3.9 sqft with 10 birds per pen distributed in 4 batteries each with 3 tiers of 6 pens/tier. Each pen is equipped with a stainless-steel feeder and one nipple drinker. Water was supplied to each pen *via* a nipple drinker system. No paper was put in the cage floor, chicken feces fall through the wired cage floor to the collection pans underneath the battery cages.

The room was environmentally controlled. Test diets and water were provided for *ad libitum* consumption throughout the experiment. The room was preheated to 27°C 2 days prior to experiment and kept at 27°C from 0 to 14 days. Room temperature was reduced to 26°C on d 14, 24°C on d 21, and 21°C on d 24 and kept at 21°C until the end of the experiment. For the first 3 days, 23 h of light was provided. The light period was reduced to 18 h from d 4 to 12 and to 16 h on d 13 until the end of the experiment.

### Experimental diets and design

Experimental diets were formulated to meet or exceed nutritional recommendations (according to Agri Stats 2021) for broiler chickens except for TSAA for experiment #1. Composition and calculated nutrient profile of starter and grower diets in two experiments were shown in Tables 1, 2. For each phase, a common basal diet was made to reduce variation among test diets from weighing and mixing; aliquots of the basal diet were then supplemented with different sources of minerals. The methionine contribution from 2-hydroxy-4-methylthiobutyric acid (HMTBa) in MMHAC was taken into consideration in both experiments. The starter diets were offered in crumbled, grower diets in pellet form with the pelleting temperature of 85°C. Xanthophylls from Oro Glo 20 (Kemin, Verona, Missouri) were added to grower diets in both experiments to increase sensitivity of serum coloration as indication of nutrient absorption and gut inflammation.

**TABLE 1:** Composition and calculated nutrient analysis of basal diets, source and dose of digestible TSAA and supplemental met in experiment #1.

Ingredient	Starter, %	Grower, %
Corn	52.65	55.53
Soybean meal	34.23	28.39
DDGS	5.00	7.50
Meat and bone meal	3.00	3.00
Choice white grease	2.55	3.27
Limestone	0.93	0.94
Salt	0.42	0.41
Dicalcium phosphate	0.31	0.09
L-Lysine HCl 78%	0.14	0.15
Threonine	0.10	0.09
Choline Cl-60%	0.05	0.05
Vitamin premix	0.05	0.05
Phytase	0.005	0.005
Oro Glo 20 (pigment)	0.10	0.10
Trace mineral premix^a^ ^,^ ^b^ ^,^ ^c^	0.20	0.20
Methionine	0.27	0.23
Total	100	100
Calculated nutrient profile		
ME, kcal/kg	3044	3126
Crude protein, %	23.23	21.32
Lys, digestible%	1.25	1.12
Met, digestible%	0.35	0.34
TSAA, digestible%	0.70	0.66
Thr, digestible%	0.88	0.80
Arg, digestible%	1.43	1.28
Val, digestible%	1.04	0.96
Trp, digestible%	0.25	0.22
Ile, digestible%	0.95	0.86
Ca, %	0.95	0.89
P, available%	0.50	0.46
P, total%	0.79	0.73
Sodium%	0.20	0.20
Analyzed proximate		
Protein, %	23.50	21.54
Dry matter, %	88.58	87.29
Ash, %	5.43	5.00
Fat, %	5.11	6.04
Fiber, %	2.99	2.59
Digestible TSAA and supplemental Met source and dose		
5% lower dig. TSAA	0.89	0.82
Suppl. DL-Met, %	0.19	0.16
Suppl. HMTBa, %	0.22	0.18
5% higher dig. TSAA	0.98	0.91
Suppl. DL-Met, %	0.29	0.25
Suppl. HMTBa, %	0.32	0.28

^a^ Trace mineral premix were included at 0.2%.

^b^ Final concentration of minerals in diets supplemented with ITM: zinc sulfate monohydrate, 80 mg/kg; Copper Sulfate, 20 mg/kg; manganese sulfate monohydrate, 100 mg/kg; sodium selenite, 0.3 mg/kg; ferrous sulfate monohydrate, 80 mg/kg; calcium iodate, 1.25 mg/kg.

^c^ Final concentration of minerals in diets supplemented with MMHAC: Zn-methionine hydroxy-analogue (Zn-MHAC), 40 mg/kg; Cu-MHAC, 10 mg/kg; Mn-MHAC, 50 mg/kg; sodium selenite, 0.3 mg/kg; ferrous sulfate monohydrate, 80 mg/kg; calcium iodate, 1.25 mg/kg.

**TABLE 2:** Composition and calculated nutrient analysis of test diets in experiment #2.

Ingredients	Starter %	Grower %
Corn	55.41	62.65
Soybean meal	34.61	27.72
Meat and bone meal	3.00	3.00
DDGS	2.50	2.50
Soy oil	1.95	2.02
Limestone	0.80	0.74
Salt	0.42	0.43
Dicalcium phosphate	0.37	0.11
MHA - 84%	0.22	0.16
Trace mineral premix^a^ ^,^ ^b^ ^,^ ^c^	0.20	0.20
L-LYSINE HCL 78%	0.14	0.14
Threonine	0.10	0.04
Oro Glo	0.10	0.10
CHOLINE CL-60%	0.05	0.06
Vitamin premix	0.05	0.05
Phytase	0.015	0.015
Total	100.0	100.0
Calculated nutrient profile		
ME, kcal/kg	3044	3120
Crude protein, %	22.93	20.09
Lys, digestible%	1.25	1.08
Met, digestible%	0.59	0.51
TSAA, digestible%	0.94	0.82
Thr, digestible%	0.88	0.72
Arg, digestible%	1.43	1.24
Val, digestible%	1.03	0.92
Trp, digestible%	0.25	0.21
Ile, digestible%	0.95	0.83
Ca, %	0.95	0.84
P, available%	0.50	0.44
P, total%	0.79	0.71
Sodium%	0.20	0.20
Analyzed proximate		
Protein, %	22.83	20.67
Dry matter, %	87.96	87.56
Ash, %	5.49	4.74
Fat, %	4.68	4.66
Fiber, %	2.28	2.40

^a^ Trace mineral premix were included at 0.2%.

^b^ Final concentration of minerals in diets supplemented with ITM: zinc sulfate monohydrate, 110 mg/kg; manganese sulfate monohydrate (MnSO_4_ H2O), 120 mg/kg; TBCC, 125 mg/kg; sodium selenite, 0.3 mg/kg; ferrous sulfate monohydrate, 80 mg/kg; calcium iodate, 1.25 mg/kg.

^c^ Final concentration of minerals in diets supplemented with MMHAC: Zn-methionine hydroxy-analogue (Zn-MHAC), 40 mg/kg; Cu-MHAC, 30 mg/kg; Mn-MHAC, 40 mg/kg; sodium selenite, 0.3 mg/kg; ferrous sulfate monohydrate, 80 mg/kg; calcium iodate, 1.25 mg/kg.

In experiment #1, a total of 720 day-old Ross 308 male broilers were randomly assigned to one of the 9 treatments of 2 × 2 × 2 factorial design + 1 arrangement with main factors of Met sources [(DL-Met vs. 2-hydroxy-4-(methylthio)-butanoic acid (HMTBa)], total sulfur amino acid (TSAA) levels (high vs. low; ±5% of recommended level. −5% TSAA was achieved by supplementation of 20% less Met, from either DL-Met or HMTBa to achieve 0.89% and 0.82% TSAA in starter diet and grower diet, respectively. +5% TSAA was achieved by supplementation of 20% more Met, from either DL-Met or HMTBa to achieve 98% and 0.91%TSAA in starter diet and grower diet, respectively), and sources of trace minerals (TM) Zn, Cu, and Mn in the form of sulfates (80:20:100 ppm) vs. MMHAC (MINTREX^®^ Zn:Cu:Mn, 40:10:50 ppm, Novus International, Inc.). Basal diet in treatment 9 was 5% more TSAA from HMTBa and TM from MMHAC (Table 1). All birds except for treatment 9 were orally gavaged with 1×, 4× and 16× the recommended dose of coccidiosis vaccine (mixed species of *E. acervulina*, *E. tenella*, and *E. maxima* from Huvepharma, Inc.) on d0, d7 and d14, respectively. Each diet was fed to 8 replicate pens of 10 broilers with randomized complete block design. Birds were fed starter diet from d0 to d13 and grower diets from d14 to d29. Growth performance was recorded at day 0, 7, 14, 20, and 27. Feed conversion ratio (FCR) was adjusted for mortality. Cumulative Performance Index (cPI) was calculated as (cumulative livability*body weight*100/day of experiment)/cumulative FCR.

In experiment #2, a total of 720 day-old Ross 308 male broilers were randomly assigned to one of the 2 dietary treatments—inorganic trace minerals (ITM) [ZnSO_4_:tribasic copper chloride (TBCC):MnSO_4_ 110:125:120 ppm] and MMHAC (Zn:Cu:Mn 40:30:40 ppm), each with 36 replicate pens of 10 birds in a randomized complete block design. All final test diets were formulated to contain the same amount of supplemental HMTBa (Table 2). All birds were orally gavaged with 1×, 4× and 16× the recommended dose of coccidiosis vaccine (mixed species of *E. acervulina*, *E. tenella*, and *E. maxima* from Huvepharma, Inc.) on d0, d7 and d14, respectively. Birds were fed starter diet from d0 to d13 and grower diets from d14 to d28. Growth performance was recorded at day 0, 7, 14, 20, and 28. On d20, 1 bird/pen was sacrificed to collect serum to measure serum coloration. On d28, 1 bird/pen was sacrificed to collect serum to measure serum coloration, α1-acid glycoprotein, and IFNγ, and jejunal tissue to quantify gut health associated gene expression.

### Analytical analysis of experimental diets

Dietary proximate analyses and amino acid quantification were performed at a commercial laboratory (Agricultural Experimental Station Chemical Laboratories, University of Missouri, Columbia, MO) according to 2006 AOAC Official Method 934.01 (moisture), Method 990.03 (crude protein), Method 920.39 (A) (crude fat), Method 978.10, 2006 (crude fiber), Method 942.05 (ash), and Method 982.30 E (a, b, c) (amino acids). Dietary total P, Ca, and Na were analyzed by ICP according to AOAC Method 965.17/865.01 mod (Eurofins Scientific Inc. Des Moines, IA). Trace minerals Zn, Cu and Mn were measured by Inductively coupled plasma-optical emission spectrometry (ICP-OES). Supplemental Met was analyzed by Hitachi amino acid analyzer after extraction from diet samples using hydrochloric acid. Supplemental HMTBa was analyzed by HPLC-UV after extraction from diet samples using 10% acetonitrile and hydrolysis.

### Sample collection

In experiment #2, at 21 and 28 d-of-age, one representative chicken with average body weight from each cage was euthanized by CO_2_ asphyxiation for sample collection. Blood sample was taken from cardiac puncture using a syringe, kept at room temperature for 3 h to allow clotting, and centrifuged (1,000 × g for 15 min) to separate serum. One cm section of jejunum was washed with cold PBS buffer and stored in 1.2 ml RNALater (Thermo Fisher Scientific Inc. Rockford, IL, United States) using 2 ml tube for 24 h at 4°C and then stored at −20°C until total ribonucleic acid (RNA) extraction for quantitation of gene expression.

### Serum coloration, α1-acid glycoprotein and interferon gamma determination

Serum coloration was measured at optical density at 480 nm in 100 μl serum using clear 96-well plate using BIO-TEK ELx800 (BIO-TEK Instrument, Winooski, VT). α1-acid glycoprotein and IFNγ were measured by ELISA using chicken α1-acid glycoprotein measurement kit from the Institute for Metabolic Ecosystem (Miyagi, Japan) and FineTest (Wuhan, China), respectively, according to the manufacturing procedure.

### Quantitation of gene expression by quantitative real time polymerase chain reaction

Total RNA was isolated from jejunum using Trizol reagent (Thermo Fisher Scientific Inc. Rockford, IL, United States). One µg of total RNA was used to synthesize complementary DNA using oligo dT and M-MLV Reverse Transcriptase (Thermo Fisher Scientific Inc. Rockford, IL, United States) according to the manufacturers’ instructions. Relative levels of mRNA were measured by qRT-PCR using Applied Biosystems SYBR Green PCR Master Mix (Thermo Fisher Scientific Inc. Rockford, IL, United States) and a QS5 Real-Time PCR System. Results were expressed as the level relative to the corresponding housekeeping gene *beta*-*actin*. The Ct of housekeeping gene *beta*-*actin* was not statistically affected by dietary treatment. Therefore *beta-actin* was chosen as housekeeping gene for this experiment and quantified along with each gene, and relative expression of each gene was normalized to *beta-actin* using delta-delta-Ct method as described previously (Livak and Schmittgen, 2001) and expressed as the level relative to *beta-actin*. All primers (Table 3) were verified for melting curve, efficiency (100% ± 10%), and linearity (r^2^ =0.99) of amplification.

**TABLE 3:** The sequence of primers for qRT-PCR.

Genes	Forward primer	Reverse primer	Accession number	Fragment size (bp)
β-actin	CAA​CAC​AGT​GCT​GTC​TGG​TGG​TA	ATC​GTA​CTC​CTG​CTT​GCT​GAT​CC	L08165	205
IL-17A	GCT​GGA​TGC​CTA​ACC​CAA​AA	TCC​TGG​TTC​ATG​TTG​CTG​ATG	NM_204460.2	79
IL1β	CAGCCCGTGGGCATCA	CTT​AGC​TTG​TAG​GTG​GCG​ATG​TT	NM_204524	58
IFNγ	AGC​TCC​CGA​TGA​ACG​ACT​TG	AGA​CTG​GCT​CCT​TTT​CCT​TTT​GA	FJ538003	102

IL-17A: interleukin 17A; IL1β, interleukin 1β; IFNγ: Interferon γ

### Data analysis

Data were analyzed as randomized complete block design with block as a random effect using the PROC MIXED procedure of SAS 9.4. Pen was the experiment unit for all analysis. Data from experiment #1 were subject to one-way (all treatments) and three-way (excluding un-challenged control) ANOVA; means were separated by Dunnett’s test to determine whether treatments were different from the un-challenged control for one-way ANOVA and means were separated by Fisher’s protected LSD test for three-way ANOVA. Data from experiment #2 were analyzed by two-sample t-test. *p*-value ≤ 0.05 was considered statistically different.

## Results

### Analysis of experimental diets

The analytical analysis of diets confirmed that moisture, crude protein, crude fat, crude fiber, amino acids, P, Ca, Na, Zn, Cu, Mn, and TSAA, which include Met, HMTBa and Cys, of all diets were as expected indicating proper mixing of test diets was achieved. The analyzed concentration of Zn, Cu, Mn and TSAA in experiment #1 was shown in Table 4. The analyzed concentration of Zn, Cu, Mn in experiment #2 was shown in Table 5.

**TABLE 4:** Formulated and analyzed Zn, Cu, Mn concentrations, and TSAA% in starter and grower diets in experiment #1.

	Formulated Zn, Cu, Mn and TSAA%								
	T1	T2	T3	T4	T5	T6	T7	T8	T9
Zn, ppm	80	80	40	40	80	80	40	40	40
Cu, ppm	20	20	10	10	20	20	10	10	10
Mn, ppm	100	100	50	50	100	100	50	50	50
TSAA% in starter diet	0.89	0.89	0.89	0.89	0.98	0.98	0.98	0.98	0.98
TSAA% in grower diet	0.82	0.82	0.82	0.82	0.91	0.91	0.91	0.91	0.91
	Analyzed Zn, Cu, Mn and TSAA in starter diet								
	T1	T2	T3	T4	T5	T6	T7	T8	T9
Zn, ppm	106.0	138.0	72.4	88.1	146.0	107.0	77.6	89.6	81.3
Cu, ppm	24.6	24.6	17.5	15.4	27.5	27.9	22.6	15.8	15.4
Mn, ppm	86.3	105.0	72.0	72.5	88.8	105.0	78.2	81.6	79.3
TSAA, %	0.66	0.87	0.87	0.89	1.03	0.95	1.01	0.98	0.97
	Analyzed Zn, Cu, Mn and TSAA in grower diet								
	T1	T2	T3	T4	T5	T6	T7	T8	T9
Zn, ppm	94.9	134.0	73.8	86.1	93.6	104.0	83.9	59.5	65.6
Cu, ppm	24.8	27.9	16.0	18.1	26.9	26.2	16.3	12.7	17.6
Mn, ppm	98.6	96.3	78.6	63.4	94.7	111.0	97.0	77.9	64.2
TSAA, %	0.85	0.81	0.78	0.79	0.95	0.90	0.92	0.88	0.87

**TABLE 5:** Formulated and analyzed Zn, Cu, Mn concentrations in starter and grower diets in experiment #2.

	Formulated concentration (ppm)		
Treatment	Zn	Cu	Mn
ITM	110	125	120
MMHAC	40	30	40
	Analyzed concentration (ppm) in starter diet		
Treatment	Zn	Cu	Mn
ITM	127.2	141.5	136.7
MMHAC	72.1	50.2	62.4
	Analyzed concentration (ppm) in grower diet		
Treatment	Zn	Cu	Mn
ITM	117.8	97.0	83.5
MMHAC	72.1	36.5	51.2

### Experiment #1

Factorial analysis showed that there was no three-way Met*TM*TSAA interaction. TM and TSAA had significant main effects on growth performance parameters, but source of Met did not impact on growth performance in this experiment (Tables 6, 7). MMHAC significantly (*p* < 0.05) improved body weight (BW) on d7, 14, and 27, cumulative feed intake (cFI) on d7, 14, 20 and 27 and cumulative feed conversion ratio (cFCR) on d27, and numerically improved (*p* < 0.10) BW on d20 in comparison to high levels of ITM (Tables 6, 7). High dose of TSAA significantly (*p* < 0.05) improved BW, cFI, and cPI at all time points, and numerically improved (*p* < 0.10) cFCR on d20 Tables 6, 7. There was no difference of mortality except for higher mortality when MMHAC and low-TSAA were fed to birds on d14, which was not continued to the end of the study, d27 (Tables 6, 7). There were no two-way interactions except for the mineral*TSAA interaction in cPI on d7 where cPI was improved only when both MMHAC and high dose of TSAA were used (Tables 6, 7).

**TABLE 6:** Factorial analysis of growth performance of birds at 7 and 14 d of age in experiment #1.

	d7					d14				
	BW	cFCR	cFI	cMort	cPI	BW	cFCR	cFI	cMort	cPI
DL-Met	0.174	1.006	0.136	1.563	243.4	0.472	1.166	0.503	1.875	282.9
HMTBa	0.173	1.011	0.135	1.250	241.7	0.466	1.174	0.499	2.188	276.6
SEM	0.002	0.004	0.001	0.619	3.2	0.008	0.005	0.007	0.761	5.7
ITM	0.171 ^b^	1.012	0.133 ^b^	0.938	239.4	0.460 ^b^	1.173	0.493 ^b^	1.563	275.0
MMHAC	0.176 ^a^	1.004	0.137 ^a^	1.875	245.8	0.478 ^a^	1.167	0.510 ^a^	2.500	284.4
SEM	0.002	0.004	0.001	0.619	3.2	0.008	0.005	0.007	0.761	5.7
Low TSAA	0.171 ^b^	1.008	0.133 ^b^	1.875	237.5 ^b^	0.459 ^b^	1.175	0.492 ^b^	2.813	270.5 ^b^
High TSAA	0.176 ^a^	1.008	0.138 ^a^	0.938	247.6 ^a^	0.479 ^a^	1.164	0.511 ^a^	1.250	289.0 ^a^
SEM	0.002	0.004	0.001	0.619	3.2	0.008	0.005	0.007	0.761	5.7
DL-Met: ITM	0.171	1.009	0.133	1.250	239.6	0.461	1.170	0.492	1.875	275.0
DL-Met: MMHAC	0.177	1.003	0.138	1.875	247.3	0.483	1.162	0.514	1.875	290.7
HMTBa: ITM	0.171	1.016	0.133	0.625	239.1	0.460	1.176	0.493	1.250	275.1
HMTBa: MMHAC	0.175	1.006	0.136	1.875	244.2	0.472	1.171	0.506	3.125	278.1
SEM	0.002	0.006	0.002	0.876	4.6	0.010	0.007	0.009	1.076	7.3
ITM: Low TSAA	0.171 ^b^	1.012	0.132	0.625	239.7 ^b^	0.454	1.182	0.488	1.250 ^b^	270.3
ITM: High TSAA	0.172 ^b^	1.013	0.134	1.250	239.0 ^b^	0.467	1.164	0.497	1.875 ^ab^	279.8
MMHAC: Low TSAA	0.171 ^b^	1.005	0.133	3.125	235.3 ^b^	0.464	1.169	0.495	4.375 ^a^	270.7
MMHAC: High TSAA	0.181 ^a^	1.004	0.141	0.625	256.2 ^a^	0.491	1.164	0.524	0.625 ^b^	298.2
SEM	0.002	0.006	0.002	0.876	4.6	0.010	0.007	0.009	1.076	7.3
DL-Met: Low TSAA	0.171	1.004	0.132	1.875	237.3	0.459	1.166	0.488	1.875	274.9
DL-Met: High TSAA	0.178	1.007	0.139	1.250	249.6	0.486	1.166	0.519	1.875	290.9
HMTBa: Low TSAA	0.172	1.012	0.133	1.875	237.7	0.459	1.184	0.496	3.750	266.1
HMTBa: High TSAA	0.174	1.010	0.136	0.625	245.7	0.472	1.163	0.503	0.625	287.1
SEM	0.002	0.006	0.002	0.876	4.6	0.010	0.007	0.009	1.076	7.3
DL-Met: ITM: Low TSAA	0.169	1.008	0.130	1.250	236.1	0.450	1.171	0.479	1.250	269.6
DL-Met: ITM: High TSAA	0.174	1.009	0.136	1.250	243.1	0.473	1.168	0.506	2.500	280.4
DL-Met: MMHAC: Low TSAA	0.172	1.000	0.134	2.500	238.5	0.468	1.161	0.496	2.500	280.1
DL-Met: MMHAC: High TSAA	0.182	1.006	0.142	1.250	256.0	0.499	1.163	0.532	1.250	301.3
HMTBa: ITM: Low TSAA	0.173	1.015	0.135	0.000	243.3	0.458	1.192	0.497	1.250	271.1
HMTBa: ITM: High TSAA	0.169	1.018	0.132	1.250	234.9	0.461	1.161	0.489	1.250	279.1
HMTBa: MMHAC: Low TSAA	0.170	1.009	0.132	3.750	232.0	0.461	1.176	0.494	6.250	261.2
HMTBa: MMHAC: High TSAA	0.180	1.002	0.140	0.000	256.5	0.484	1.166	0.517	0.000	295.0
SEM	0.003	0.008	0.003	1.239	6.4	0.012	0.010	0.011	1.522	9.9
*p* value										
Met	0.5514	0.3862	0.5940	0.7226	0.6987	0.4138	0.2969	0.5649	0.7725	0.3456
Mineral	0.0255	0.1734	0.0364	0.2891	0.1662	0.0309	0.3817	0.0181	0.3874	0.1609
TSAA	0.0192	0.9677	0.0066	0.2891	0.0305	0.0135	0.1442	0.0087	0.1521	0.0070
Met*Mineral	0.6110	0.6510	0.4561	0.7226	0.7853	0.5303	0.8915	0.5242	0.3874	0.3395
Mineral*TSAA	0.0470	0.8655	0.0738	0.0799	0.0210	0.3703	0.3771	0.1720	0.0469	0.1769
Met*TSAA	0.3039	0.6608	0.2295	0.7226	0.6458	0.3703	0.1659	0.0911	0.1521	0.7102
Met*Mineral*TSAA	0.3039	0.5444	0.2036	0.2891	0.2244	0.6967	0.5685	0.4271	0.3874	0.5606

Different superscript letters ^a,b^ show significant differences (*p* ≤ 0.05).

**TABLE 7:** Factorial analysis of growth performance of birds at 20 and 27 d of age in experiment #1.

	d20					d27				
	BW	cFCR	cFI	cMort	cPI	BW	cFCR	cFI	cMort	cPI
DL-Met	0.853	1.262	1.024	2.686	327.8	1.540	1.339	1.984	1.899	420.4
HMTBa	0.839	1.268	1.012	1.749	324.3	1.534	1.334	1.985	1.503	421.7
SEM	0.011	0.005	0.010	1.167	6.3	0.021	0.008	0.021	1.386	11.1
ITM	0.831	1.269	1.004 ^b^	1.124	322.9	1.500 ^b^	1.350 ^a^	1.948 ^b^	0.649	411.5
MMHAC	0.860	1.261	1.033 ^a^	3.311	329.2	1.573 ^a^	1.322 ^b^	2.020 ^a^	2.753	430.6
SEM	0.011	0.005	0.010	1.1674	6.3	0.021	0.0083	0.0208	1.386	11.1
Low TSAA	0.826 ^b^	1.272	0.999 ^b^	2.999	314.2 ^b^	1.510 ^b^	1.341	1.948 ^b^	2.753	407.8 ^b^
High TSAA	0.866 ^a^	1.258	1.038 ^a^	1.436	337.9 ^a^	1.564 ^a^	1.332	2.020 ^a^	0.649	434.2 ^a^
SEM	0.011	0.005	0.010	1.167	6.3	0.021	0.008	0.021	1.386	11.1
DL-Met: ITM	0.838	1.265	1.008	1.436	325.0	1.507	1.355	1.948	0.649	411.6
DL-Met: MMHAC	0.868	1.259	1.040	3.936	330.6	1.573	1.322	2.020	3.149	429.1
HMTBa: ITM	0.825	1.274	0.999	0.811	320.7	1.494	1.345	1.949	0.649	411.3
HMTBa: MMHAC	0.853	1.263	1.026	2.686	327.8	1.573	1.322	2.021	2.356	432.0
SEM	0.015	0.008	0.015	1.498	8.9	0.028	0.012	0.029	1.681	13.7
ITM: Low TSAA	0.816	1.277	0.990	0.811	316.3	1.479	1.356	1.913	0.649	403.8
ITM: High TSAA	0.846	1.261	1.017	1.436	329.5	1.522	1.345	1.984	0.649	419.2
MMHAC: Low TSAA	0.836	1.267	1.007	5.186	312.1	1.541	1.326	1.983	4.856	411.9
MMHAC: High TSAA	0.885	1.255	1.059	1.436	346.2	1.605	1.319	2.057	0.649	449.3
SEM	0.015	0.008	0.015	1.498	8.9	0.028	0.012	0.029	1.681	13.7
DL-Met: Low TSAA	0.827	1.267	0.995	2.686	316.9	1.509	1.345	1.938	1.899	410.2
DL-Met: High TSAA	0.878	1.258	1.053	2.686	338.7	1.571	1.333	2.030	1.899	430.5
HMTBa: Low TSAA	0.825	1.278	1.002	3.311	311.5	1.511	1.337	1.959	3.606	405.4
HMTBa: High TSAA	0.853	1.258	1.022	0.186	337.0	1.556	1.331	2.011	0.000	438.0
SEM	0.015	0.008	0.015	1.498	8.9	0.028	0.012	0.029	1.681	13.7
DL-Met: ITM: Low TSAA	0.819	1.271	0.989	0.811	318.5	1.482	1.370	1.909	0.024	402.9
DL-Met: ITM: High TSAA	0.856	1.260	1.028	2.061	331.6	1.531	1.340	1.986	1.274	420.3
DL-Met: MMHAC: Low TSAA	0.834	1.263	1.002	4.561	315.2	1.536	1.319	1.966	3.774	417.6
DL-Met: MMHAC: High TSAA	0.901	1.255	1.079	3.311	345.9	1.610	1.325	2.073	2.524	440.7
HMTBa: ITM: Low TSAA	0.814	1.284	0.992	0.811	314.1	1.476	1.342	1.917	1.274	404.7
HMTBa: ITM: High TSAA	0.836	1.263	1.006	0.811	327.4	1.513	1.349	1.981	0.024	418.0
HMTBa: MMHAC: Low TSAA	0.837	1.272	1.013	5.811	309.0	1.546	1.332	2.000	5.937	406.1
HMTBa: MMHAC: High TSAA	0.869	1.254	1.039	0.000	346.6	1.600	1.313	2.041	0.000	457.9
SEM	0.021	0.011	0.021	2.000	12.5	0.039	0.017	0.042	2.152	17.8
*p* value										
Met	0.3625	0.4348	0.4174	0.4826	0.6912	0.8125	0.6837	0.9633	0.7690	0.9071
Mineral	0.0573	0.2685	0.0490	0.1047	0.4806	0.0080	0.0198	0.0178	0.1236	0.0973
TSAA	0.0104	0.0609	0.0103	0.2438	0.0099	0.0479	0.4456	0.0173	0.1236	0.0234
Met*Mineral	0.9467	0.7730	0.8821	0.8146	0.9321	0.8213	0.6720	0.9961	0.7690	0.8882
Mineral*TSAA	0.5184	0.8367	0.3961	0.1047	0.2428	0.7036	0.8541	0.9556	0.1236	0.3337
Met*TSAA	0.4218	0.5052	0.1983	0.2438	0.8382	0.7563	0.7797	0.5005	0.1236	0.5878
Met*Mineral*TSAA	0.7448	0.9539	0.6690	0.4826	0.8519	0.9455	0.1837	0.6587	0.5285	0.4735

Different superscript letters ^a,b^ show significant differences (*p* ≤ 0.05).

Dunnett’s test was used to compare the growth performance of challenged birds with different nutritional supplements [Treatment (T) 1–8)] to un-challenged birds (T9) and the results were shown in Tables 8, 9. Compared to un-challenged birds (T9), growth performance of *Eimeria*-challenged birds was substantially reduced with higher cFCR on d7, 14 and 20 and lower BW and cFI on d14, 20 and 27. In addition, the BW and cPI of birds on d27 in T7 and T8 were not significantly different from unchallenged birds (T9), indicating that supplementation of both MMHAC and high dose of TSAA improved the BW and cPI of birds subjected to *Eimeria* challenge to the similar level as un-challenged birds regardless of Met source at 27 d of age.

**TABLE 8:** One way Anova analysis of growth performance of birds at 7 and 14 d in experiment #1.

Treatment	BW	cFCR	cFI	cMort	cPI
d7					
T1: DL-Met (5% Low TSAA) + ITM	0.169*	1.008*	0.130*	1.25	236.1*
T2, HMTBa (5% Low TSAA) + ITM	0.173	1.015*	0.135	0.00	243.3
T3: DL-Met (5% Low TSAA) + MMHAC	0.173	1.000*	0.134	2.50	238.5*
T4: HMTBa (5% Low TSAA) + MMHAC	0.170*	1.009*	0.132*	3.75	232.0*
T5: DL-Met (5% High TSAA) + ITM	0.174	1.009*	0.136	1.25	243.1
T6: HMTBa (5% High TSAA) + ITM	0.169*	1.017*	0.132*	1.25	234.9*
T7: DL-Met (5% High TSAA) + MMHAC	0.182	1.006*	0.142	1.25	256.0
T8: HMTBa (5% High TSAA) + MMHAC	0.180	1.002*	0.140	0.00	256.5
T9: HMTBa (5% High TSAA) + MMHAC, no challenge	0.180	0.976	0.140	1.25	259.5
SEM	0.003	0.008	0.002	1.24	6.3
*p* value	0.0141	0.0488	0.0084	0.5430	0.0135
d14					
T1: DL-Met (5% Low TSAA) + ITM	0.444*	1.171*	0.473*	1.25	267.7*
T2: HMTBa (5% Low TSAA) + ITM	0.453*	1.192*	0.491*	1.25	269.2*
T3: DL-Met (5% Low TSAA) + MMHAC	0.463*	1.161*	0.491*	2.50	278.3*
T4: HMTBa (5% Low TSAA) + MMHAC	0.455*	1.176*	0.488*	6.25	259.4*
T5: DL-Met (5% High TSAA) + ITM	0.467*	1.168*	0.500	2.50	278.6*
T6: HMTBa (5% High TSAA) + ITM	0.456*	1.161*	0.483*	1.25	277.3*
T7: DL-Met (5% High TSAA) + MMHAC	0.494	1.163*	0.526	1.25	299.5
T8: HMTBa (5% High TSAA) + MMHAC	0.478	1.166*	0.511	0.00	293.2
T9: HMTBa (5% High TSAA) + MMHAC, no challenge	0.506	1.124	0.524	1.25	317.4
SEM	0.011	0.011	0.010	1.49	9.1
*p* value	0.0016	0.0054	0.0030	0.2087	0.0007

*Statistical different (*p* ≤ 0.05) from un-challenged control (T9) by Dunnett’s test.

**TABLE 9:** Oneway ANOVA analysis of growth performance of birds at 20 and 27 d in experiment #1.

Treatment	BW	cFCR	cFI	cMort	cPI
d20					
T1: DL-Met (5% Low TSAA) + ITM	0.819*	1.271*	0.989*	1.25	318.5*
T2: HMTBa (5% Low TSAA) + ITM	0.814*	1.284*	0.992*	1.25	314.1*
T3: DL-Met (5% Low TSAA) + MMHAC	0.835*	1.263*	1.002*	5.00	315.2*
T4: HMTBa (5% Low TSAA) + MMHAC	0.837*	1.272*	1.013*	6.25	309.0*
T5: DL-Met (5% High TSAA) + ITM	0.856*	1.260*	1.028*	2.50	331.6*
T6: HMTBa (5% High TSAA) + ITM	0.836*	1.263*	1.006*	1.25	327.4*
T7: DL-Met (5% High TSAA) + MMHAC	0.901	1.255*	1.079	3.75	345.9*
T8: HMTBa (5% High TSAA) + MMHAC	0.869*	1.254*	1.039*	0.00	346.6*
T9: HMTBa (5% High TSAA) + MMHAC, no challenge	0.953	1.215	1.109	1.25	387.2
SEM	0.020	0.010	0.020	1.82	12.0
*p* value	<0.0001	0.0029	0.0003	0.2660	0.0003
d27					
T1: DL-Met (5% Low TSAA) + ITM	1.477*	1.370	1.906*	1.25	395.3*
T2: HMTBa (5% Low TSAA) + ITM	1.471*	1.342	1.917*	2.50	397.0*
T3: DL-Met (5% Low TSAA) + MMHAC	1.532*	1.319	1.966*	5.00	409.9*
T4: HMTBa (5% Low TSAA) + MMHAC	1.534*	1.332	1.990*	7.14	397.7*
T5: DL-Met (5% High TSAA) + ITM	1.527*	1.34	1.986*	2.50	412.6*
T6: HMTBa (5% High TSAA) + ITM	1.508*	1.349	1.981*	1.25	410.3*
T7: DL-Met (5% High TSAA) + MMHAC	1.606	1.325	2.073	3.75	433.0
T8: HMTBa (5% High TSAA) + MMHAC	1.595	1.313	2.041*	0.00	450.3
T9: HMTBa (5% High TSAA) + MMHAC, no challenge	1.671	1.303	2.166	1.25	461.6
SEM	0.036	0.016	0.042	1.86	15.8
*p* value	0.0011	0.1542	0.0002	0.2313	0.0246

*Statistical different (*p* ≤ 0.05) from un-challenged control (T9) by Dunnett’s test.

### Experiment #2

MMHAC supplementation improved cFCR and cPI without affecting cFI at all time points with 2.1 points improvement of cFCR on d28, and increased body weight by 37 g on d28 in comparison to high dose of ITM, and there was no difference of mortality between the two treatments at any time points (Table 10).

**TABLE 10:** Growth performance of birds at 7, 14, 20, and 28 d in experiment #2.

Treatment	Body weight (kg)	cFCR	cFI (kg)	cperfidx
d7				
ITM	0.202	1.011^a^	0.159	283.5^b^
MMHAC	0.202	0.996^b^	0.157	290.5^a^
SEM	0.001	0.003	0.001	2.0
*p* value	0.9433	0.0006	0.2172	0.0149
d14				
ITM	0.542	1.184^a^	0.588	323.3^b^
MMHAC	0.547	1.170^b^	0.587	333.1^a^
SEM	0.004	0.004	0.004	2.5
*p* value	0.3260	0.0020	0.8560	0.0063
d20				
ITM	1.042	1.261^a^	1.258	407.7^b^
MMHAC	1.052	1.252^b^	1.261	419.0^a^
SEM	0.006	0.004	0.007	3.3
*p* value	0.2269	0.0388	0.7249	0.0149
d28				
ITM	1.870^b^	1.372^a^	2.503	482.6^b^
MMHAC	1.907^a^	1.351^b^	2.508	502.9^a^
SEM	0.010	0.005	0.012	4.5
*p* value	0.0161	0.0001	0.7569	0.0007

Different superscript letters ^a,b^ show significant differences (*p* ≤ 0.05).

**FIGURE 1:**
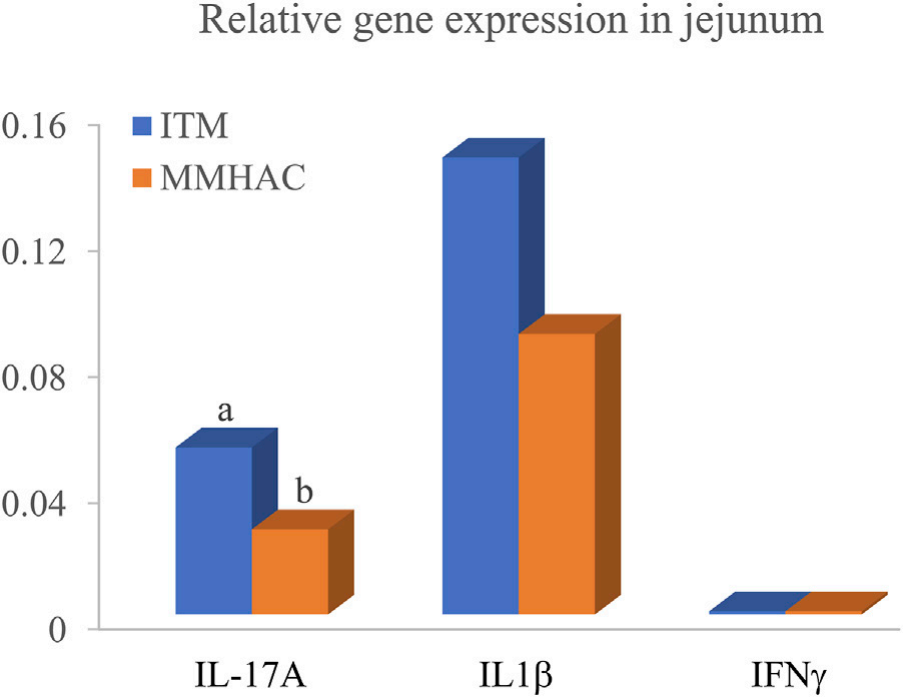
Jejunal gene expression of birds at 28 d in experiment #2. Different superscript letters ^a,b^ show significant differences (*p* ≤ 0.05).

MMHAC numerically (*p* < 0.10) improved serum coloration on d20 and d28 by 9.9% and 9.1%, respectively (Table 11). Although serum α1-acid glycoprotein and IFNγ concentrations were not statistically different on d28, α1-acid glycoprotein and IFNγ were slightly lower with MMHAC supplementation (Table 11). Jejunal IL-17A gene expression was significantly (*p* = 0.046) reduced by 49% in MMHAC treatment on d28 (Figure 1). Although jejunal IL1β and IFNγ gene expression was not statistically different on d28, IL1β gene expression was numerically (*p* = 0.1177) reduced by 39% in MMHAC treatment (Figure 1).

**TABLE 11:** Serum parameters of birds at 20 and 28 d in experiment #2.

Treatment	Serum		
	Coloration	α1-acid glycoprotein	IFNγ
d20			
ITM	0.493		
MMHAC	0.542		
SEM	0.019		
*p* value	0.0805		
d28			
ITM	0.693	139.300	0.708
MMHAC	0.756	125.144	0.600
SEM	0.026	6.676	0.068
*p* value	0.0939	0.1396	0.2700

## Discussion

TSAA (Met + Cys) are essential amino acids in corn/soybean meal-based diets. Some research has shown that chickens infected with *Eimeria* may need higher amount of TSAA (Southern and Baker, 1982). Increase of dietary Met level from 0.45% to 0.56% and 0.68% improved growth performance of broilers mediated with narasin, an anticoccidial drug, but not in vaccinated chickens (Lai et al., 2018). Inclusion of additional dietary TSAA by adding 0.3% and 0.4% DL-Met, which resulted in 0.9% and 1.0% standardized ileal digestible (SID) of TSAA, respectively, did not mitigate the growth suppression caused by *Eimeria* infection in comparison to 0.1% DL-Met (0.8% SID of TSAA, Ren et al., 2020). Both 0.73%% and 0.88% of TSAA by supplementation of 0.30% and 0.45% L-Met, respectively, increased body weight gain and feed intake to the similar extent compared to 0.58% of TSAA in broilers regardless of *Eimeria* challenge, indicating that 0.73% TSAA was enough to maintain growth performance of broilers with or without *Eimeria* challenge (Castro et al., 2020). These findings indicated that the requirements of TSAA for chickens infected with *Eimeria* are inconsistent. In the experiment #1, high level of TSAA (0.98% in starter diet and 0.91% in grower diet, ∼105% of recommended level) improved body weight and cumulative feed intake compared to low level of TSAA (0.89% in starter diet and 0.82% in grower diet, ∼95% of recommended level) regardless of adding HMTBa or DL-Met. It’s possible that the broilers with multiple rounds of *Eimeria* infection, which is similar to the occurrence of coccidiosis in the field, had lower digestibility and/or absorption of TSAA and had to activate their immune system to fight against coccidiosis, therefore, higher amount of Met is required to meet the need of TSAA to maintain growth and support the immune response to pathogenic *Eimeria* species.

In experiment #1, compared to high dose of ZnSO_4_:CuSO_4_:MnSO_4_, low dose of MMHAC had significant main effect in improving body weight, feed intake and FCR. In experiment #2, low dose of MMHAC improved FCR during all phases and body weight at 28 d of age without effect on feed intake in comparison to high dose of ZnSO_4_:TBCC:MnSO_4_. The Cu inclusion level in MMHAC treatment in experiment#2 was higher than that in experiment #1, the better growth performance from MMHAC supplementation than ITM with high dose of TBCC suggests that MMHAC has higher efficacy than ITM in both sulfate and TBCC form in improving growth performance of broilers under coccidiosis. In both experiments, MMHAC had greater magnitude improvement of body weight and/or FCR during recovery phase (d20-28), suggesting that MMHAC probably promoted the recovery of broilers from coccidia challenge. Similar to these two studies, feeding Zn amino acid complex improved FCR in young broilers at 10 d of age (de Grande et al., 2020), and feeding methionine chelated Zn and Mn blended with inorganic source of minerals increased body weight in large white male turkeys at 18 weeks of age (Flores et al., 2021). MMHAC has been reported to improve antioxidant status in broilers (Richards et al., 2010; Bun et al., 2011; Qi et al., 2019), on the other hand, inorganic Cu especially when supplemented at high levels could become pro-oxidants and increase reactive oxygen species and malondialdehyde leading to oxidative stress (Yang et al., 2019). Inclusion of MMHAC in broiler diets reduced oxidative stress by decreasing blood lipid hydroperoxides levels (Richards et al., 2010). Supplementation of 80 mg/kg Zn-MHAC in aged laying hens improved Cu/Zn superoxide dismutase activity in liver and serum and total antioxidant capacity in liver (Qi et al., 2019). Zn-MHAC supplementation increased the activities of plasma Cu-Zn superoxide dismutase (Cu/Zn-SOD) and glutathione peroxidase and concentration of cecal secretory IgA in both *E. tenella*-challenged and nonchallenged birds and decreased fecal oocyst count in *E. tenella*-challenged birds, which supported that Zn-MHAC reduced oxidative stress and improved some immune responses in broilers (Bun et al., 2011). Similarly, Zn amino acid complex improved FCR during starter phase (0-10d) and duodenal morphology with greater villus height and villus height/crypt depth ratio in broilers at 10 and 28 d of age, and reduced plasma malondialdehyde concentration at only 10 d of age (de Grande et al., 2020). Inclusion of 90 or 120 mg/kg Zn glycine chelate increased activities of Cu/Zn-SOD and glutathione peroxidase and reduced malondialdehyde content in livers in broilers at 21 and 42 d of age and improved jejunal morphology at 42 d of age (Ma et al., 2011). Dietary supplementation of low dose of Mn methionine hydroxyl analog chelated improved the growth performance and antioxidant capacity compared to high dose of ITM in broilers (Meng et al., 2021). On the other hand, feeding 30% or 50% dose of organic trace minerals as inorganic trace minerals did not improve growth performance of broilers (Zhu et al., 2019), and feeding the same dose of amino acid-chelated minerals as ITM did not improve growth performance in broilers regardless of heat stress challenge although it improved gut barrier function in broilers under heat stress (Baxter et al., 2020). The improvement of growth performance by feeding low dose of MMHAC in broilers subjected to coccidia challenge in the current two studies was likely due to their higher bioavailability (Yan and Waldroup, 2006; Wang et al., 2007; Richards et al., 2015), greater antioxidant capacity and enhanced immune modulation of MMHAC than ITM, which remains to be confirmed.

Plasma levels of carotenoids are often used as nutritional indicators for their dietary intake (Schweigert, 2001). Enteric infections and inflammation can decrease the absorption of many nutrients including dietary carotenoids (Rubin et al., 2017). Both inflammation and nutritional deficiency could cause poor absorption of carotenoids leading to a reduction of serum carotenoids, therefore reduction of serum carotenoids could indicate either inflammation or reduced nutrient intake or both. The magnitude of the decrease in serum carotenoids is often proportional to the severity of disease (Schweigert, 2001). *E. acervulina* infection caused pale-bird syndrome, and a substantial loss of previously absorbed carotenoids along with drastic malabsorption of dietary carotenoids in broilers (Tyczkowski et al., 1991). *E. acervulina* challenge decreased plasma carotenoids and reduced growth performance and apparent ileal digestibility of amino acids in a dose-dependent manner (Rochel et al., 2016). Serum coloration measured at 480 nm**,** which is produced exclusively by carotenoid pigments in the feed, has been reported as a criterion of the severity of experimental coccidiosis in chicken, it decreases with the increase of *Eimeria* doses, and correlate**s** more closely with the severity of infection with *E. maxima* or *E. acervulina* than with *E. tenella* (Yvore et al., 1993). Zhao et al., observed that *E. acervulina* infection reduced serum carotenoid levels at 7 days post infection, *E. tenella* infection decreased serum carotenoid levels at 14 days post infection, and dietary zinc amino acid complex increased serum carotenoid levels in *E. tenella*-infected birds but not in *E. maxima*-infected birds (Zhao et al., 2015). In experiment #2, MMHAC numerically (*p* < 0.1) increased serum coloration in mixed *Eimeri*a species (*E. acervuline/E. maxima/E. tenella*)-infected birds. Since there was no difference in feed intake, the numerical increase of serum coloration indicates that MMHAC likely numerically reduced inflammation caused by *Eimeria* challenge.

IL-17A is produced by Th17 cells and plays a critical role in mediating inflammation during early infection and prior to the onset of adaptive T cell responses against infectious pathogens in a variety of *in vivo* mammalian infection model systems (Steinman, 2007; Chen and O’Shea, 2008; McGeachy and Cua, 2008; Ivanov et al., 2009; Iwakura et al., 2011). IL-17 could synergize with tumor necrosis factor-a (TNF-a) and IL-1β to increase the proinflammatory responses (Min et al., 2013). Recently a few studies described IL-17 family of cytokines as a key mediator of immunity in avian coccidiosis (Min and Lillehoj, 2002; Hong et al., 2008; Kim et al., 2012). Hong et al. (2006a) and Hong et al. (2006b), observed that IL-17A mRNA levels were remarkably increased in intestinal intraepithelial lymphocytes by up to 2,020-fold at 5 days post infection of *E. acervulina* and 1,650-fold at 4 days post-infection of *E. maxima*. Zhang et al. (2013) reported that IL-17, IL1β and IL6 were increased in cecal intraepithelial lymphocytes during early infection of *E. tenella*, peaking at 6 d post injection and declining thereafter, and treating chickens with IL-17 neutralized antibody reduced fecal oocyst shedding and cecal lesion scores and enhanced body weight gain probably by reducing the production of IL-17, IL6 and TGFβ. These findings suggest that IL-17 likely mediated the immunopathology induced by *Eimeria* infection. Similarly, Del Cacho et al. (2014) used IL-17A neutralizing antibody to counteract IL-17A bioactivity in *E. tenella*-infected chickens and found that the IL-17A neutralizing antibody decreased cecal lesion scores and reactive oxygen species production and reduced intracellular schizont and merozoite development. These studies support that IL-17A might be a potential therapeutic target for coccidiosis control. In experiment #2, MMHAC significantly reduced jejunal IL-17A gene expression, and numerically reduced jejunal IL1β gene expression and serum α1-acid glycoprotein concentration. These results suggest that MMHAC might have down-regulated immunopathology caused by the series of *Eimeria* vaccine challenge, thereby numerically reduced jejunal and systemic inflammation, which is consistent with the tendency of less inflammation indicated by numerical increase of serum coloration and could be part of the reasons MMHAC improved growth performance of broilers under *Eimeria* challenge.

In summary, high levels of TSAA significantly improved growth performance compared to low levels of TSAA and Met source did not impact growth performance in broilers subjected to *Eimeria* challenge, indicating that broilers with coccidia challenge are responding to TSAA levels higher than commercial and breeder recommendations with better growth performance. Supplementation of the reduced levels of a highly bioavailable bis-chelated trace minerals in the form of MMHAC improved growth performance compared to high levels of ITM (sulfates or sulfate combined with high dose of TBCC). The benefits of MMHAC might partially result from the enhanced immune response against coccidiosis due to the higher bioavailability and greater antioxidant capacity, which warrants further investigation in future studies. Feeding MMHAC and high dose of TSAA increased the growth performance of boilers under coccidia challenge to the same level as un-challenged birds, suggesting that combination of bis-chelated trace minerals MMHAC and high levels of TSAA could help broilers overcome growth performance challenge due to coccidiosis. The findings from these two experiments suggest that bis-chelated trace minerals that are chelated to methionine hydroxy-analogue, MMHAC, could compliment coccidia vaccination program to control coccidiosis in broilers by enhancing immune response against coccidiosis.

## Data Availability

The original contributions presented in the study are included in the article/Supplementary Material; further inquiries can be directed to the corresponding author.

## Abbreviations

AGP, antibiotic growth promoters; BW, body weight; cFI, cumulative feed intake; cFCR, cumulative feed conversion ratio; cPI, cumulative performance index; Cu, copper; Cys, cysteine; Cu-MHAC, Cu methionine hydroxy-analogue chelate; HMTBa, 2-hydroxy-4-(methylthio)-butanoic acid; IFNg, interferon gamma; Mn, manganese; MMHAC, mineral methionine hydroxy-analogue bis-chelate; Met, methionine; qRT-PCR, quantitative real time polymerase chain reaction; RNA, ribonucleic acid; SID, standardized ileal digestible; TBCC, tribasic copper chloride; TM, trace minerals; T, treatment; TSAA, total sulfur amino acid; Zn, zinc; Zn-MHAC, Zn methionine hydroxy-analogue chelate.

## References

[B1] Baxter M. F. A. Greene E. S. Kidd M. T. Tellez-Isaias G. Orlowski S. Dridi S. (2020). Water amino acid-chelated trace mineral supplementation decreases circulating and intestinal HSP70 and proinflammatory cytokine gene expression in heat-stressed broiler chickens. J. Anim. Sci. 98 (3), skaa049. 10.1093/jas/skaa049 32047923 PMC7070152

[B2] Bean-Hodgins L. Kiarie E. G. (2021). Mandated restrictions on the use of medically important antibiotics in broiler chicken production in Canada: Implications, emerging challenges, and opportunities for bolstering gastrointestinal function and health — A review. Can. J. Anim. Sci. 101, 602–629. 10.1139/cjas-2021-0015

[B3] Blake D. P. Knox J. Dehaeck B. Huntington B. Rathinam T. Ravipati V. (2020). Re-calculating the cost of coccidiosis in chickens. Vet. Res. 51 (1), 115. 10.1186/s13567-020-00837-2 32928271 PMC7488756

[B4] Bun S. D. Guo Y. M. Guo F. C. Ji F. J. Cao H. (2011). Influence of organic zinc supplementation on the antioxidant status and immune responses of broilers challenged with Eimeria tenella. Poult. Sci. 90 (6), 1220–1226. 10.3382/ps.2010-01308 21597062

[B5] Castro F. L. S. Tompkins Y. H. Pazdro R. Kim W. K. (2020). The effects of total sulfur amino acids on the intestinal health status of broilers challenged with Eimeria spp. Poult. Sci. 99 (10), 5027–5036. 10.1016/j.psj.2020.06.055 32988539 PMC7598302

[B6] Chapman H. D. (2009). A landmark contribution to poultry science--prophylactic control of coccidiosis in poultry. Poult. Sci. 88 (4), 813–815. 10.3382/ps.2008-00316 19276426

[B7] Chapman H. D. Jeffers T. K. (2014). Vaccination of chickens against coccidiosis ameliorates drug resistance in commercial poultry production. Int. J. Parasitol. Drugs Drug Resist. 4 (3), 214–217. 10.1016/j.ijpddr.2014.10.002 25516830 PMC4266793

[B8] Chen Z. O’Shea J. J. (2008). Th17 cells: A new fate for differentiating helper T cells. Immunol. Res. 41, 87–102. 10.1007/s12026-007-8014-9 18172584

[B9] De Grande A. Leleu S. Delezie E. Rapp C. De Smet S. Goossens E. (2020). Dietary zinc source impacts intestinal morphology and oxidative stress in young broilers. Poult. Sci. 99 (1), 441–453. 10.3382/ps/pez525 32416829 PMC7587869

[B10] Del Cacho E. Gallego M. Lillehoj H. S. Quílez J. Lillehojm E. P. Ramo A. (2014). IL-17A regulates Eimeria tenella schizont maturation and migration in avian coccidiosis. Vet. Res. 45 (1), 25. 10.1186/1297-9716-45-25 24571471 PMC3975951

[B11] El Sabry M. I. Stino F. K. R. El-Ghany W. A. A. (2021). Copper: Benefits and risks for poultry, livestock, and fish production. Trop. Anim. Health Prod. 53 (5), 487. 10.1007/s11250-021-02915-9 34590182

[B12] FASS (2010). Guide for care and use of agricultural animals in research and teaching. 3rd Edition. Champaign, IL: Federation of Animal Science Societies.

[B13] Fawcett D. W. (1994). “Bone. Pages 194–229,” in A textbook of histology. 12th ed. (New York, NY: Chapman & Hall).

[B14] FDA. (2012). Guidance for Industry #209. The judicious use of medically important antimicrobial drugs in food-producing animals.

[B15] FDA. (2013). Guidance for industry #213. New animal drugs and new animal drug combination products administered in or on medicated feed or drinking water of food-producing animals: Recommendations for drug sponsors for voluntarily aligning product use conditions with GFI #209.

[B16] FDA. (2015). Veterinary feed directive.

[B17] Flores K. R. Fahrenholz A. Ferket P. R. Biggs T. J. Grimes J. L. (2021). Effect of methionine chelated Zn and Mn and corn particle size on Large White male Turkey live performance and carcass yields. Poult. Sci. 100 (11), 101444. 10.1016/j.psj.2021.101444 34547618 PMC8463767

[B18] Hong Y. H. Lillehoj H. S. Dalloul R. A. Min W. Miska K. B. Tuo W. (2006a). Molecular cloning and characterization of chicken NKlysin. Vet. Immunol. Immunopathol. 110, 339–347. 10.1016/j.vetimm.2005.11.002 16387367

[B19] Hong Y. H. Lillehoj H. S. Lee S. H. Dalloul R. A. Lillehoj E. P. (2006b). Analysis of chicken cytokine and chemokine gene expression following Eimeria acervulina and Eimeria tenella infections. Vet. Immunol. Immunopathol. 114, 209–223. 10.1016/j.vetimm.2006.07.007 16996141

[B20] Hong Y. H. Lillehoj H. S. Park D. W. Lee S. H. Han J. Y. Shin J. H. (2008). Cloning and functional characterization of chicken interleukin-17D. Vet. Immunol. Immunopathol. 126 (1-2), 1–8. 10.1016/j.vetimm.2008.06.002 18649949

[B21] Ivanov I. Atarashi K. Manel N. Brodie E. L. Shima T. Karaoz U. (2009). Induction of intestinal Th17 cells by segmented filamentous bacteria. Cell 139, 485–498. 10.1016/j.cell.2009.09.033 19836068 PMC2796826

[B22] Iwakura Y. Ishigame H. Saijo S. Nakae S. (2011). Functional specialization of interleukin-17 family members. Immunity 34 (2), 149–162. 10.1016/j.immuni.2011.02.012 21349428

[B23] Kim W. H. Jeong J. Park A. R. Yim D. Kim Y. H. Kim K. D. (2012). Chicken IL-17F: Identification and comparative expression analysis in eimeria-infected chickens. Dev. Comp. Immunol. 38, 401–409. 10.1016/j.dci.2012.08.002 22922588

[B24] Koh T. S. Peng R. K. Klasing K. C. (1996). Dietary copper level affects copper metabolism during lipopolysaccharide-induced immunological stress in chicks. Poult. Sci. 75 (7), 867–872. 10.3382/ps.0750867 8805205

[B25] Lai A. Dong G. Song D. Yang T. Zhang X. (2018). Responses to dietary levels of methionine in broilers medicated or vaccinated against coccidia under Eimeria tenella-challenged condition. BMC Vet. Res. 14 (1), 140. 10.1186/s12917-018-1470-8 29699573 PMC5922021

[B26] Lekshmi M. Ammini P. Kumar S. Varela M. F. (2017). The food production environment and the development of antimicrobial resistance in human pathogens of animal origin. Microorganisms 5 (1), 11. 10.3390/microorganisms5010011 28335438 PMC5374388

[B27] Li C. Guo S. Gao J. Guo Y. Du E. Lv Z. (2015). Maternal high-zinc diet attenuates intestinal inflammation by reducing DNA methylation and elevating H3K9 acetylation in the A20 promoter of offspring chicks. J. Nutr. Biochem. 26 (2), 173–183. 10.1016/j.jnutbio.2014.10.005 25541535

[B28] Li G. Q. Kanu S. Xiao S. M. Xiang F. Y. (2005). Responses of chickens vaccinated with a live attenuated multi-valent ionophore-tolerant Eimeria vaccine. Vet. Parasitol. 129 (3-4), 179–186. 10.1016/j.vetpar.2004.09.034 15845272

[B29] Livak K. J. Schmittgen T. D. (2001). Analysis of relative gene expression data using real-time quantitative PCR and the 2(-Delta Delta C(T)) Method. Methods 25 (4), 402–408. 10.1006/meth.2001.1262 11846609

[B30] Ma W. Niu H. Feng J. Wang Y. Feng J. (2011). Effects of zinc glycine chelate on oxidative stress, contents of trace elements, and intestinal morphology in broilers. Biol. Trace Elem. Res. 142 (3), 546–556. 10.1007/s12011-010-8824-9 20734240

[B31] Martín-Venegas R. Brufau M. T. Guerrero-Zamora A. M. Mercier Y. Geraert P. A. Ferrer R. (2013). The methionine precursor DL-2-hydroxy-(4-methylthio)butanoic acid protects intestinal epithelial barrier function. Food Chem. 141 (3), 1702–1709. 10.1016/j.foodchem.2013.04.081 23870881

[B32] Matsushita K. Takahashi K. Yukio A. (2007). Effects of adequate or marginal excess of dietary methionine hydroxy analogue free acid on growth performance, edible meat yields and inflammatory response in female broiler chickens. J. Poult. Sci. 44, 265–272. 10.2141/jpsa.44.265

[B33] McGeachy M. J. Cua D. J. (2008). Th17 cell differentiation: The long and winding road. Immunity 28 (4), 445–453. 10.1016/j.immuni.2008.03.001 18400187

[B34] Meng T. Gao L. Xie C. Xiang Y. Huang Y. Zhang Y. (2021). Manganese methionine hydroxy analog chelated affects growth performance, trace element deposition and expression of related transporters of broilers. Anim. Nutr. 7 (2), 481–487. 10.1016/j.aninu.2020.09.005 34258436 PMC8245798

[B35] Min W. Kim W. H. Lillehoj E. P. Lillehoj H. S. (2013). Recent progress in host immunity to avian coccidiosis: IL-17 family cytokines as sentinels of the intestinal mucosa. Dev. Comp. Immunol. 41 (3), 418–428. 10.1016/j.dci.2013.04.003 23583525

[B36] Min W. Lillehoj H. S. (2002). Isolation and characterization of chicken interleukin-17 cDNA. J. Interferon Cytokine Res. 22 (11), 1123–1128. 10.1089/10799900260442548 12513911

[B37] Peek H. W. Landman W. J. (2011). Coccidiosis in poultry: Anticoccidial products, vaccines and other prevention strategies. Vet. Q. 31 (3), 143–161. 10.1080/01652176.2011.605247 22029884

[B38] Qi X. Ma S. Liu X. Wang Y. Liu Y. Gao Y. (2019). Effects of the methionine hydroxyl analogue chelate zinc on antioxidant capacity and liver metabolism using ^1^H-NMR-Based metabolomics in aged laying hens. Animals. 9 (11), 898. 10.3390/ani9110898 31683848 PMC6912617

[B39] Ren Z. Bütz D. E. Whelan R. Naranjo V. Arendt M. K. Ramuta M. D. (2020). Effects of dietary methionine plus cysteine levels on growth performance and intestinal antibody production in broilers during Eimeria challenge. Poult. Sci. 99 (1), 374–384. 10.3382/ps/pez503 32416822 PMC7587792

[B40] Richards J. D. Fisher P. M. Evans J. L. Wedekind K. J. (2015). Greater bioavailability of chelated compared with inorganic zinc in broiler chicks in the presence or absence of elevated calcium and phosphorus. Open Access Anim. Physiol. 7, 97–110. 10.2147/OAAP.S83845

[B41] Richards J. D. Zhao J. Harrell R. J. Atwell C. A. Dibner J. J. (2010). Trace mineral nutrition in poultry and swine. Asian-Australas. J. Anim. Sci. 23 (11), 1527–1534. 10.5713/ajas.2010.r.07

[B42] Richards M. P. Augustine P. C. (1988). Serum and liver zinc, copper, and iron in chicks infected with Eimeria acervulina or Eimeria tenella. Biol. Trace Elem. Res. 17, 207–219. 10.1007/BF02795457 2484359

[B43] Rochel S. J. Parsons C. M. Dilger R. N. (2016). Effects of Eimeria acervulina infection severity on growth performance, apparent ileal amino acid digestibility, and plasma concentrations of amino acids, carotenoids, and α1-acid glycoprotein in broilers. Poult. Sci. 95 (7), 1573–1581. 10.3382/ps/pew035 26933234

[B44] Rubin L. P. Ross A. C. Stephensen C. B. Bohn T. Tanumihardjo S. A. (2017). Metabolic effects of inflammation on vitamin A and carotenoids in humans and animal models. Adv. Nutr. 8 (2), 197–212. 10.3945/an.116.014167 28298266 PMC5347109

[B45] Schweigert F. J. (2001). Inflammation-induced changes in the nutritional biomarkers serum retinol and carotenoids. Curr. Opin. Clin. Nutr. Metab. Care 4 (6), 477–481. 10.1097/00075197-200111000-00002 11706279

[B46] Southern L. L. Baker D. H. (1982). Eimeria acervulina infection in chicks fed excess copper in the presence or absence of excess dietary methionine. J. Anim. Sci. 54 (5), 989–997. 10.2527/jas1982.545989x 7096222

[B47] Steinman L. (2007). A brief history of T(H)17, the first major revision in the T(H)1/T(H)2 hypothesis of T cell-mediated tissue damage. Nat. Med.Nat Med. 1313 (23), 139385–140145. 10.1038/nm1551 17290272

[B48] Surai P. F. Earle-Payne K. Kidd M. T. (2021). Taurine as a natural antioxidant: From direct antioxidant effects to protective action in various toxicological models. Antioxidants (Basel) 10 (12), 1876. 10.3390/antiox10121876 34942978 PMC8698923

[B49] Tang K. L. Caffrey N. P. Nóbrega D. B. Cork S. C. Ronksley P. E. Barkema H. W. (2017). Restricting the use of antibiotics in food-producing animals and its associations with antibiotic resistance in food-producing animals and human beings: A systematic review and meta-analysis. Lancet. Planet. Health 1 (8), e316–e327. 10.1016/S2542-5196(17)30141-9 29387833 PMC5785333

[B50] Teng P. Y. Choi J. Yadav S. Tompkins Y. H. Kim W. K. (2021). Effects of low-crude protein diets supplemented with arginine, glutamine, threonine, and methionine on regulating nutrient absorption, intestinal health, and growth performance of Eimeria-infected chickens. Poult. Sci. 100 (11), 101427. 10.1016/j.psj.2021.101427 34551373 PMC8463775

[B51] Tyczkowski J. K. Hamilton P. B. Ruff M. D. (1991). Altered metabolism of carotenoids during pale-bird syndrome in chickens infected with Eimeria acervulina. Poult. Sci. 10, 2074–2081. 10.3382/ps.0702074 1956852

[B52] Underwood E. J. Suttle N. F. (1999). The mineral nutrition of livestock. 3rd Edition. Wallingford, Oxon: CABI Publishing, 283–292. 10.1079/9780851991283.0283

[B53] Wang Y. Yin X. Yin D. Lei Z. Mahmood T. Yuan J. (2019). Antioxidant response and bioavailability of methionine hydroxy analog relative to DL-methionine in broiler chickens. Anim. Nutr. 5 (3), 241–247. 10.1016/j.aninu.2019.06.007 31528725 PMC6737507

[B54] Wang Z. Cerrate S. Coto C. Yan F. Waldroup P. W. (2007). Evaluation of Mintrex_®_ copper as a source of copper in broiler diets. Int. J. Poult. Sci. 6 (5), 308–313. 10.3923/ijps.2007.308.313

[B55] Willemsen H. Swennen Q. Everaert N. Geraert P. A. Mercier Y. Stinckens A. (2011). Effects of dietary supplementation of methionine and its hydroxy analog DL-2-hydroxy-4-methylthiobutanoic acid on growth performance, plasma hormone levels, and the redox status of broiler chickens exposed to high temperatures. Poult. Sci. 90 (10), 2311–2320. 10.3382/ps.2011-01353 21934015

[B56] Xiao M. Mi Y. Liu L. Lv C. Zeng W. Zhang C. (2018). Taurine regulates mucosal barrier function to alleviate lipopolysaccharide-induced duodenal inflammation in chicken. Amino Acids 50 (11), 1637–1646. 10.1007/s00726-018-2631-6 30132121

[B57] Yan F. Waldroup P. W. (2006). Evaluation of Mintrex manganese as a source of manganese for young broilers. Int. J. Poult. Sci. 5, 708–713. 10.3923/ijps.2006.708.713

[B58] Yang F. Pei R. Zhang Z. Liao J. Yu W. Qiao N. (2019). Copper induces oxidative stress and apoptosis through mitochondria-mediated pathway in chicken hepatocytes. Toxicol. Vitro 54, 310–316. 10.1016/j.tiv.2018.10.017 30389602

[B59] Yvore P. Mancassola R. Naciri M. Bessay M. (1993). Serum coloration as a criterion of the severity of experimental coccidiosis in the chicken. Vet. Res. 24 (3), 286–290. 8343813

[B60] Zhang H. Pan S. Zhang K. Michiels J. Zeng Q. Ding X. (2020). Impact of dietary manganese on intestinal barrier and inflammatory response in broilers challenged with Salmonella typhimurium. Microorganisms 8 (5), 757. 10.3390/microorganisms8050757 32443502 PMC7285304

[B61] Zhang L. Liu R. Song M. Hu Y. Pan B. Cai J. (2013). Eimeria tenella: Interleukin 17 contributes to host immunopathology in the gut during experimental infection. Exp. Parasitol. 133 (2), 121–130. 10.1016/j.exppara.2012.11.009 23201216

[B62] Zhao X. J. Li Z. P. Wang J. H. Xing X. M. Wang Z. Y. Wang L. (2015). Effects of chelated Zn/Cu/Mn on redox status, immune responses and hoof health in lactating Holstein cows. J. Vet. Sci. 16 (4), 439–446. 10.4142/jvs.2015.16.4.439 26040614 PMC4701736

[B63] Zhu Z. Yan L. Hu S. An S. Lv Z. Wang Z. (2019). Effects of the different levels of dietary trace elements from organic or inorganic sources on growth performance, carcass traits, meat quality, and faecal mineral excretion of broilers. Arch. Anim. Nutr. 73 (4), 324–337. 10.1080/1745039X.2019.1620050 31192701

